# Model refinement increases confidence levels and clinical agreement when commissioning a three‐dimensional secondary dose calculation system

**DOI:** 10.1002/acm2.13590

**Published:** 2022-04-07

**Authors:** Brian Bismack, Jennifer Dolan, Eric Laugeman, Anant Gopal, Ning Wen, Indrin Chetty

**Affiliations:** ^1^ Henry Ford Health System Detroit Michigan USA

**Keywords:** 3D Gamma, dose comparison, model

## Abstract

**Purpose:**

Evaluate custom beam models for a second check dose calculation system using statistically verifiable passing criteria for film analysis, DVH, and 3D gamma metrics.

**Methods:**

Custom beam models for nine linear accelerators for the Sun Nuclear Dose Calculator algorithm (SDC, Sun Nuclear) were evaluated using the AAPM‐TG119 test suite (5 Intensity Modulated Radiation Therapy (IMRT) and 5 Volumetric Modulated Arc Therapy (VMAT) plans) and a set of clinical plans. Where deemed necessary, adjustments to Multileaf Collimator (MLC) parameters were made to improve results. Comparisons to the Analytic Anisotropic Algorithm (AAA), and gafchromic film measurements were performed. Confidence intervals were set to 95% per TG‐119. Film gamma criteria were 3%/3 mm (conventional beams) or 3%/1 mm (Stereotactic Radiosurgery [SRS] beams). Dose distributions in solid water phantom were evaluated based on DVH metrics (e.g., D95, V20) and 3D gamma criteria (3%/3 mm or 3%/1 mm). Film passing rates, 3D gamma passing rates, and DVH metrics were reported for HD MLC machines and Millennium MLC Machines.

**Results:**

For HD MLC machines, SDC gamma film agreement was 98.76% ± 2.30% (5.74% CL) for 6FFF/6srs (3%/1 mm), and 99.80% ± 0.32% (0.83% CL) for 6x (3%/3 mm). For Millennium MLC machines, film passing rates were 98.20% ± 3.14% (7.96% CL), 99.52% ± 1.14% (2.71% CL), and 99.69% ± 0.82% (1.91% CL) for 6FFF, 6x, and 10x, respectively. For SDC to AAA comparisons: HD MLC Linear Accelerators (LINACs); DVH point agreement was 0.97% ± 1.64% (4.18% CL) and 1.05% ± 2.12% (5.20% CL); 3D gamma agreement was 99.97% ± 0.14% (0.30% CL) and 100.00% ± 0.02% (0.05% CL), for 6FFF/6srs and 6x, respectively; Millennium MLC LINACs: DVH point agreement was 0.77% ± 2.40% (5.47% CL), 0.80% ± 3.40% (7.47% CL), and 0.07% ± 2.15% (4.30% CL); 3D gamma agreement was 99.97% ± 0.13% (0.29% CL), 99.97% ± 0.17% (0.36% CL), and 99.99% ± 0.06% (0.12% CL) for 6FFF, 6x, and 10x, respectively.

**Conclusion:**

SDC shows agreement well within TG119 CLs for film and redundant dose calculation comparisons with AAA. In some models (SRS), this was achieved using stricter criteria. TG119 plans can be used to help guide model adjustments and to establish clinical baselines for DVH and 3D gamma criteria.

## 1 INTRODUCTION

The standard for treatment planning system (TPS) calculation verification has been a point dose monitor unit calculation. Though this is often accompanied by a planar dose measurement used to assess the deliverability of the plan (patient‐specific quality assurance, PSQA), the point dose approach has limited application for modern complex treatments, as it simplifies patient geometry and heterogeneities, and does not quantitatively assess the overall dose distribution. Single‐point dose methods do not evaluate the plan's quality via metrics that are relevant to the plan's clinical effectiveness, such as target dose coverage or organ at risk sparing.^1^, ^2^ Dose–volume histogram (DVH) metrics, and therefore plan quality optimization, can also be impacted by factors such as dose grid resolution, interpolation method between dose grid points, and by the method in which structures themselves sample the dose grid.^1^, ^3^, ^4^ Considering the short comings of the current standard, it is desirable to implement second check systems that match the complexities of primary TPSs, but with the goals of speed and automation of calculation and evaluation.^2^, ^5^

The SunCHECK system (Sun Nuclear Corp., Melbourne, FL, USA) is a quality assurance (QA) system capable of three‐dimensional (3D) dose calculations on the patient Computed Tomography (CT) dataset using their Sun Nuclear Dose Calculator (SDC), a model‐based collapsed cone convolution (CCC) superposition algorithm.^6^ The SDC explicitly models the rounded leaf end as well as other factors such as a tongue‐and‐groove thickness (a parameter that has been shown to improve accuracy of Multileaf Collimator (MLC) modeling^7^).

SunCHECK has multiple modules that employ the SDC using different input data. DoseCHECK will take the original plan file from the TPS and use SDC to calculate the dose on the patient CT dataset, allowing point dose, DVH, and 3D‐gamma comparisons (region of interest specific and overall body) to the primary TPS dose calculation. PerFRACTION has a pretreatment QA module and a “during‐treatment” QA module, both of which use SDC to estimate delivered 3D dose from MLC trajectory log files or from MLC positions derived from CINE electronic portal imaging device (EPID) imaging. Additionally, PerFRACTION can employ SDC by taking integrated EPID images (either in air pretreatment imaging or transit dose imaging during treatment) and performing planar dose calculations; allowing for a means of EPID‐measured planar dose QA.^8^

Additional features such as automatic primary TPS dose grid matching, user‐defined clinical DVH goals, 3D visualizations of dose, 3D gamma agreement distributions, and custom reporting options round out the tools of the platform.

In this paper, we evaluate the performance of the SDC under controlled systematic commissioning conditions. The processes, data, and experience conveyed in this paper outline a framework that will aid in the rigor and speed of commissioning these more complex second check and PSQA systems.

## 2 MATERIALS AND METHODS

Institutions work in conjunction with Sun Nuclear to develop beam models for their systems. The beam modeling process was an iterative process and close collaboration with the vendor enabled finely tuned refinement of beam models. Film and ion chamber measurements were acquired, and each machine's SDC model was compared to calculations using the Analytical Anisotropic Algorithm (AAA) of our primary TPS (Eclipse, Varian Medical Systems). Models for beam energies across nine linear accelerators were evaluated. Linear accelerator platforms included the TrueBeam (Varian Medical System, Palo Alto, CA, USA) and the Trilogy employing either the Millenium MLC or HD MLC systems (including the Varian Edge system^9^). Aggregate analysis for an instance of each MLC machine type is located in the Appendix allowing readers to reference various equipment setups they may encounter in the clinic. Much of the modeling work is performed by Sun Nuclear, however institutions are able to provide guidance for modeling tradeoffs or discrepancies noted between measurements and calculations.

The modeling is done in a two‐phase process. The first phase is primarily concerned with open beam data modeling: matching profiles, percent depth dose curves (PDDs), output factors, and surface buildup. Sun Nuclear requests open field plans and calculated doses for typical field sizes (2 × 2 cm^2^, 5 × 5 cm^2^, 10 × 10 cm^2^, 20 × 20 cm^2^, and 40 × 40 cm^2^) in a water phantom, machine calibration conditions, a CT–HU curve, and an output factor table. Sun Nuclear utilizes a base model that starts with an average beam data set and adjusts various modeling parameters to better match data measured by the institution. Small fields (2 × 2 cm^2^ or 3 × 3 cm^2^) are used to check the primary spectrum and slope of the geometric penumbra. This serves as a check on the focal source size parameter, which can be adjusted to match the dose fall off near the field edge.

The output factor table is then used to verify the output factors of the various field sizes of the base model. SDC starts with an output factor prediction based on its model, then compares it to the measured output factors and employs a correction factor to match its calculated values to the measured. The goal is for the correction factors to be within ±1%, achieved by adjusting the radius and weighting parameters of the extra focal source. If the correction factors are beyond ±1%, Sun Nuclear informs the site and recommends an investigation of the site's TPS open‐field commissioning before proceeding further.

The shoulders of the 40 × 40 cm^2^ field help check if the institution is properly modeling the collimator at extreme scenarios. This can be clinically impactful for treatments that are attempting to treat larger gross anatomy within one field, such as attempting to treat the whole femur at extended Source to Skin Distances (SSDs) to help streamline the treatment workflow.

SDC uses a polyenergetic kernel that is a weighted average of various energy‐binned monoenergetic kernels.^10^ The relative weighting of the monoenergetic kernels is determined by the photon energy spectrum (which gets broken up into corresponding energy bins). Thus, the photon spectrum of the model needs to be verified. This is done using PDDs of the various field sizes, specifically using the region beyond *d*_max_ to avoid electron contamination effects. Typically, modification is not needed as the spectra are similar between machines of the same model.

The second phase of modeling deals with fine tuning the MLC model with the intent to better match verified delivered plans, a method that has shown improvement in predicted dose/deliverability in other dose calculation systems such as Eclipse or Mobius 3D (Mobius Medical System, Houston, TX, USA).^11^, ^12^ SDC does not use a Dosimetric Leaf Gap (DLG) value, but instead explicitly models the MLC leaf end.^6^ There are four major MLC parameters used by the SDC: leaf radius of curvature, leaf transmission, tongue and groove thickness, and leaf gap offset. In this case, Sun Nuclear asks for patient plan datasets. In the event matching is not achieved using well‐defined and narrow ranges for the four parameters, sweeping gap calculations from your primary TPS (gap widths of 2, 4, 6, 10, 14, 16, and 20 mm sweeping across a 10 × 10 cm^2^ field for 100 MU in a solid water phantom 30 × 30 × 20 cm^3^, 90 SSD, 10 cm depth), the corresponding sweeping gap measurements, and an L‐shaped MLC plan measurement can be requested.

The sweeping gap measurements/calculations help to tune the leaf gap offset parameter and the radius of curvature parameter, each of which are very sensitive to this measurement. The radius of curvature parameter is tuned within an especially tight window with adjustments mainly being made to the leaf offset parameter. Sweeping gaps also help with the leaf transmission, as the reading includes portions of the plan, where the ion chamber (or various points of interest) is behind MLC leaves. An ion chamber reading behind closed MLC leaves was also provided to more directly model the MLC transmission.

The tongue and groove thickness parameter can be initially set using an L‐shaped MLC plan to start. Clinical plans on patient datasets and/or phantoms can then be used to further fine tune this parameter. In our case, the L‐plan was not employed as it was not yet being recommended but it is now often being used for developing new machine models.

The vendor has a range of typical values for each of these parameters and will automatically adjust the model to match within this range. Adjustments outside of this range will require consultation with the institution for direction or inquiries as to why the performance of the beam model in question is outside of what is normally expected. Typically, adjustments are initially made on previously verified patient datasets. If satisfactory agreement was not achieved or there were problems noticed during evaluation of the models the sweeping gap data was then used to help fine tune the MLC parameters.

Calculated plans employed a universal Hounsfield Unit‐Electron Density (HU‐ED) curve that has been verified across all CT machines in the system. These machines are all from the Brilliance Big Bore (Philips) line of CT scanners. The SunCHECK system can accommodate this with an assigned default HU‐ED curve. Multiple CT machines with separate HU‐ED curves can be designated. The SunCHECK system can read the DICOM label on the CT dataset to ascertain the exact machine.

Finalized models were evaluated following recommendations from AAPM TG‐53 and TG‐119.^13^, ^14^ The evaluation primarily entailed open beam comparisons and pair‐wise comparisons of statistical agreement between SDC calculations, AAA calculations, and ion chamber/planar film measurements.

Additionally, an assessment of DVH and 3D gamma comparisons between the AAA and SDC calculated dose distributions was performed in TG119 solid water plans. This evaluation was done with the goal of establishing an expected agreement in solid water commissioning plans between the two dose calculation systems. All of the described comparisons were performed on a pilot machine, and an abbreviated process was developed for other machines in the hospital system.

### 2.1 Evaluation metrics

#### 2.1.1 Digital TG‐53 comparisons

Table 4–4 in the TG‐53 report recommends evaluation of various regions of open fields such as the inner beam, penumbra, and out‐of‐field regions. Here, we performed a digital comparison between a commissioned AAA model and the given SDC model.^13^

The inner beam is the central high dose portion of the beam. In our case, we focus on comparisons along central axis (CAX). The assessment here included square fields of 1 × 1–20 × 20 cm^2^ (2 × 2–20 × 20 cm^2^ for conventional beams) at depths ranging from 2 to 15 cm. The square fields were bound by either jaws or MLCs. In the case of MLC‐defined square fields, the jaws were set to 20 × 20 cm^2^. Percentage difference criteria were used to quantify the agreement.

TG‐53 defines the penumbra region as 0.5 cm inside and outside the projection of the edge of the defining collimator.^13^ For these profile comparisons, the dose gradient at the location of the penumbra was estimated to be linear between two points within the penumbra region. Data were selected from two points, 0.25 cm inside and outside the defining collimator, to generate a slope of the dose gradient. This slope was then employed to find the distance to agreement (DTA) from the calculated dose difference between AAA and SDC.

The out‐of‐field region is defined as the region outside of the penumbra, where the percent difference as normalized to the CAX dose was used.^13^

#### 2.1.2 TG‐119 comparisons (IMRT and VMAT commissioning)

**FIGURE 1:**
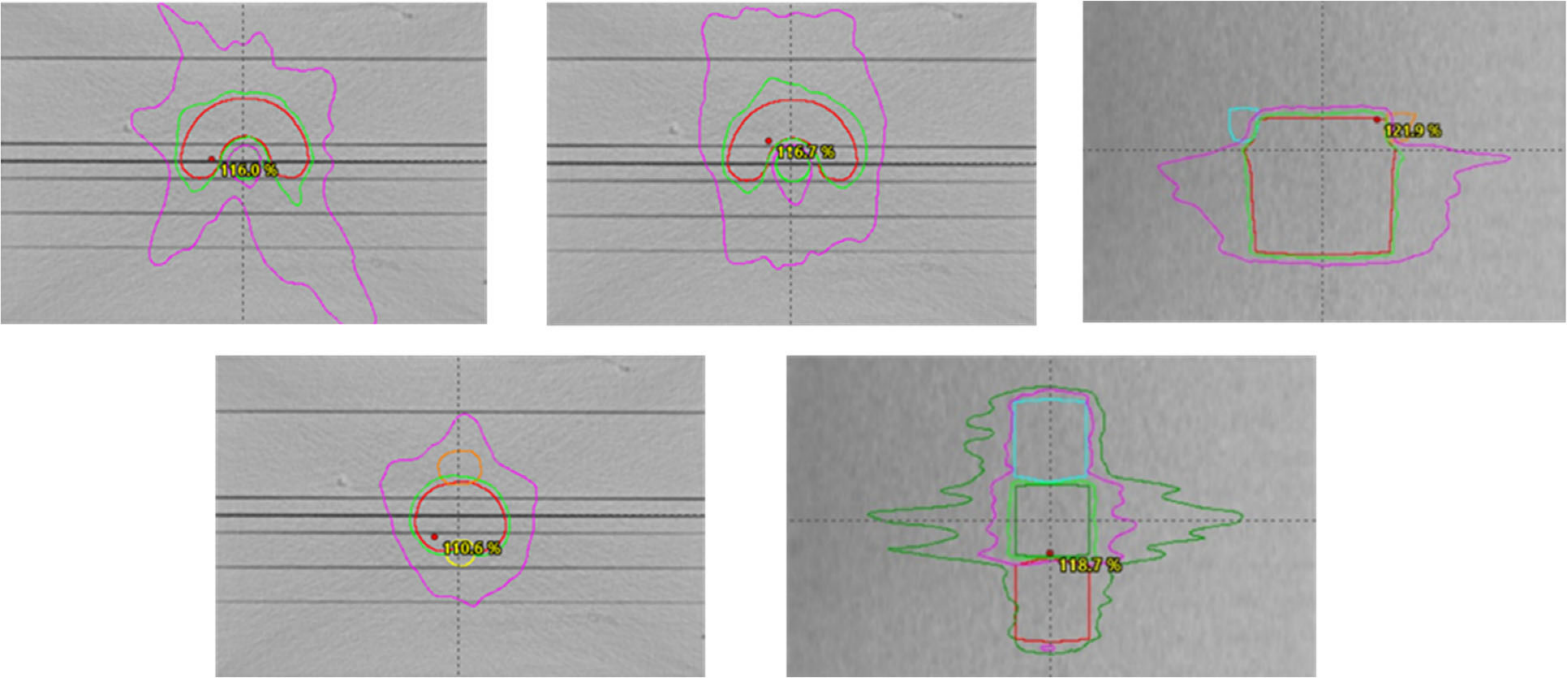
Clockwise starting from upper left: easy C‐shape (single arc, core contour in green), hard C‐shape (2 arc), head and neck (coronal view, parotids in blue and orange), prostate (orange bladder, yellow rectum), and multi‐target (dose levels in green, blue, and red in order of dose level). Isodose lines shown are 95% (light green), 50% (magenta), and 30% (dark green) of prescription dose

Guidance from TG‐119 was used to validate the SDC models (in parallel with AAA models) for each machine. For our pilot machine (TrueBeam), the 6 MV‐Flattening Filter Free (FFF) energy was selected for the most comprehensive evaluation. Ten TG‐119 plans were studied; five static gantry Intensity Modulated Radiation Therapy (IMRT) plans (using 7–9 beams) and five Rapid Arc plans (employing one‐two arcs). The plans included easy and hard “C” shapes, head and neck, prostate, and multi‐dose levels (Figure 1). Point dose measurements (ion chamber model CC01, IBA Dosimetry, Schwarzenbruck Germany) and coronal film measurements (Gafchromic EBT3, Bridgewater NJ, USA) were performed at isocenter and in the high‐dose and low‐dose regions. Additionally, film and ion chamber measurements were made at isocenter on 18 clinical plans (seven spine Stereotactic Radiosurgery [SRS], two head and neck, five lung Stereotactic Body Radiation Therapy (SBRT), two mediastinal, one abdominal, and one cranial SRS) using the same solid water setup. For the remaining beams on this machine (6x, 10x) the SDC model was evaluated with ion chamber and film measurements at isocenter for the TG‐119 plan set.

To obtain the planar dose from the SDC calculation that corresponds to the measured film, a MATLAB code was developed to locate the plane in the 3D dose file and interpolate within that plane to achieve the desired resolution. A spline interpolation was used to increase the in‐plane resolution from the native resolution (0.1 or 0.15 cm for SRS‐type plans, 0.25 cm for conventional plans) to 0.039 cm. This finer resolution level matches the in‐house film analysis method used to assess AAA treatment plans. The in‐house software package, used to analyze both AAA and SDC treatment plans, is able to streamline the process of film calibration, film scanning, dose mapping from multiple color channels, registrations, and gamma analysis.^15^ A pair‐wise comparison was then performed between AAA, SDC, and film. Gamma passing rates for film using criteria of 3%/3 mm for conventional beams (6x, 10x, etc.) and 3%/1 mm for stereotactic beams (6FFF, 6srs, etc.) were employed. Average, standard deviation, and confidence limits (CL) for a 95% confidence interval (1.96σ)^14^, ^16^ were noted. Film CL were computed using TG‐119's recommended formula (Equation (1)):

(1) $$\mathrm{CL}\;=\left(100-\mathrm{mean}\right)\;+1.96\sigma .$$

#### 2.1.3 DVH and 3D gamma comparisons

An evaluation of the agreement between the AAA calculation and the SDC calculation using clinical metrics used by the SunCHECK system was performed. This involved comparing DVH metrics and 3D gamma pass rates of dose calculations in a solid water environment using the TG‐119 plan set discussed above. DVH metrics used were D99, D95, and mean dose for targets; max point dose, D.04cc, and D.4cc for serial‐type structures; and mean, max, and various volumetric metrics (e.g., bowel V20Gy for full course or V0.48 Gy for single fraction) for parallel structures. 3D gamma pass rates were analyzed on a structure‐by‐structure basis. Criteria used were 3%/3 mm for conventional beams and 3%/1 mm for stereotactic beams.

#### 2.1.4 Abbreviated verification

For other machines/energies in the hospital system, an abbreviated process was used, focusing on validation of the models to measurement and establishing digital agreement baselines using TG‐119 plans for each machine and energy. Standardized plans for each of the above listed TG‐119 cases (five Rapid Arc and five IMRT) were copied to each machine and used for the digital comparisons. For SRS beams, a comparison between the AAA and SDC calculated doses was done with the TG‐119 plans focusing in on target coverage agreement; then, film analysis was performed for those TG‐119 plans and a selected patient plan subset; lastly, digital comparison baselines were established for the TG‐119 plans. For conventional beams, the abbreviated process focused on the TG‐119 plans, once again following the procedure of: a preliminary 3D dose grid digital comparison, film analysis (this could also be done with TG119 ion chamber measurements), and finally digital comparisons for the target and Organ at Risk (OAR) structure in the TG‐119 plans.

## 3 RESULTS

The following results are shown for the 6FFF energy on our pilot site's TrueBeam and are a digital comparison between AAA and SDC.

### 3.1 6FFF TG‐53 results

The first TG‐53 region analyzed was the CAX region (Tables 1 and 2) which recommends agreement within 1% for square jaw fields and 2% for square MLC fields. CAX percent agreement for jaw fields was within 1% at all depths for field sizes of 2 × 2 cm^2^ and above. Differences above 1% were observed for field sizes below 2 × 2 cm^2^. For MLC‐shaped fields (jaws set to 20 × 20 cm^2^), differences between SDC and AAA were correlated to depth and field size with differences greater than 2% being seen for field sizes 3 × 3 cm^2^ and below at depths of about 6 cm or greater. The largest discrepancy (roughly 3%) was observed for MLC fields below 3 × 3 cm^2^ at depths near 10 cm. This situation is particularly challenging to model as the tertiary collimator is obscuring the source more than the secondary collimator, this will strain the modeling of the extra focal source and therefore higher disagreement may be expected than for larger fields or jaw‐collimated fields. Calibration conditions (10 × 10 cm^2^ jaw positions at 10 cm depth) registered agreement within one‐hundredth of a percent. Having good agreement at calibration conditions is desirable and can serve as a check that there are no gross output errors.

**TABLE 1:** Jaw central axis readings, Analytic Anisotropic Algorithm (AAA) vs. Sun Nuclear Dose Calculator (SDC) percentage difference relative to AAA calculation. Readings are extracted from calculations on a water phantom at 100 SSD, where the beam is collimated by symmetrically set jaws to the sizes specified in the table with the MLCs fully retracted. Cells within 1% are white

Jaw field size	1 × 1	1.5 × 1.5	2 × 2	3 × 3	5 × 5	10 × 10	20 × 20
Depth/% Diff							
2cm	2.09%	1.38%	0.90%	0.65%	0.21%	–0.20%	–0.10%
3cm	1.98%	1.36%	0.72%	0.58%	0.22%	–0.32%	–0.31%
4cm	1.99%	1.06%	0.77%	0.37%	0.24%	–0.34%	–0.43%
6cm	1.98%	1.07%	0.44%	0.43%	0.27%	–0.50%	–0.71%
8cm	1.72%	0.88%	0.17%	0.00%	‐0.15%	–0.70%	–0.79%
9cm	2.06%	1.15%	0.55%	0.17%	0.33%	–0.30%	–0.42%
10cm	1.99%	1.02%	0.39%	0.00%	0.35%	0.00%	–0.15%
11cm	1.18%	0.00%	‐0.63%	‐0.80%	‐0.37%	–0.83%	–0.92%
13cm	1.36%	0.25%	‐0.24%	‐0.23%	‐0.21%	–1.13%	–0.86%
14cm	1.17%	‐0.27%	‐0.78%	‐0.25%	‐0.68%	–1.60%	–0.90%
15cm	1.89%	0.00%	‐0.28%	0.53%	‐0.49%	–1.28%	–0.57%

Abbreviation: MLC, Multileaf Collimator; SSD, Source to Skin Distance.

**TABLE 2:** MLC central axis readings, Analytic Anisotropic Algorithm (AAA) vs. Sun Nuclear Dose Calculator (SDC) percentage difference relative to AAA calculation. Readings are extracted from calculations on a water phantom at 100 SSD. MLCs were set to create a symmetric square field at the central axis with the unused MLC leaf abutment line 2 cm from the edge of the field. Jaws were set to 20 × 20 cm to isolate MLCs as collimator. Cells within 1% are white

MLC Field Size	1×1	1.5×1.5	2×2	3×3	5×5	10×10	20×20
Depth/% Diff							
2cm	−2.46%	−1.42%	−1.40%	−1.16%	−0.62%	−0.30%	−0.10%
3cm	−2.52%	−1.52%	−1.61%	−1.12%	−0.66%	−0.42%	−0.31%
4cm	−2.45%	−1.63%	−1.60%	−1.44%	−0.81%	−0.45%	−0.43%
6cm	−2.52%	−1.88%	−1.99%	−1.51%	−0.79%	−0.62%	−0.71%
8cm	−2.73%	−2.16%	−2.29%	−1.89%	−1.20%	−0.84%	−0.92%
9cm	−2.54%	−1.97%	−2.11%	−1.86%	−0.80%	−0.30%	−0.55%
10cm	−2.73%	−2.11%	−1.89%	−1.99%	−0.85%	−0.16%	−0.29%
11cm	−3.36%	−2.86%	−3.01%	−2.88%	−1.45%	−0.99%	−1.07%
13cm	−3.10%	−2.83%	−2.78%	−2.22%	−1.46%	−1.31%	−0.86%
14cm	−3.31%	−3.03%	−2.98%	−2.37%	−1.99%	−1.58%	−1.08%
15cm	−2.98%	−2.72%	−2.67%	−1.78%	−1.43%	−1.27%	−0.57%

Abbreviation: MLC, Multileaf Collimator; SSD, Source to Skin Distance.

The penumbra region (Table 3) was analyzed by examining points 0.25 cm inside and outside the defining collimator. Fields defined by jaws and MLCs were examined. For the 5 × 5 cm^2^ MLC field, jaws were set to 7 × 7 cm^2^. All results were within the TG‐53 (Table 4–4^13^) expected criteria of 2 mm.

**TABLE 3:** Distance to agreement for penumbra regions for points located at different depths, 0.25 cm inside and 0.25 cm outside the defining collimator

Field size	1 × 1 MLC		5 × 5 MLC		10 × 10 Jaw		20 × 20 Jaw	
Depth/DTA in mm	Inner	Outer	Inner	Outer	Inner	Outer	Inner	Outer
Depth/DTA in mm	Inner	Outter	Inner	Outter	Inner	Outter	Inner	Outter
*d*_max_	0.13	0.20	0.13	0.20	−0.21	−0.10	−0.04	0.33
5cm	−0.02	−0.02	−0.03	0.03	−0.05	−0.08	−0.14	0.10
10cm	−0.01	−0.01	−0.13	−0.80	−0.12	0.01	0.00	0.08

Abbreviation: MLC, Multileaf Collimator.

**TABLE 4:** Out of field % dose differences as normalized to central axis dose. Analysis included different collimation types, field sizes, distance outside of the field, and central axis depth

5x5 MLC diff			10x10 Jaw diff	
Dist outside collimator	2.5 cm	7.5 cm	Dist outside collimator	5.0cm
Depth				Depth
*d*_max_	‐0.10%	0.10%	*D*_max_	0.20%
5cm	‐0.13%	0.13%	5cm	0.00%
10cm	‐0.35%	0.00%	10cm	0.00%

Abbreviation: MLC, Multileaf Collimator.

TG‐53 recommended normalizing percent differences in the out of field region (Table 4) to the central‐ray and recommends a tolerance of 2% for jaw fields and 5% for MLC fields. Differences between SDC and AAA within 1% were observed and deemed acceptable.

### 3.2 TG‐119 results

**FIGURE 2:**
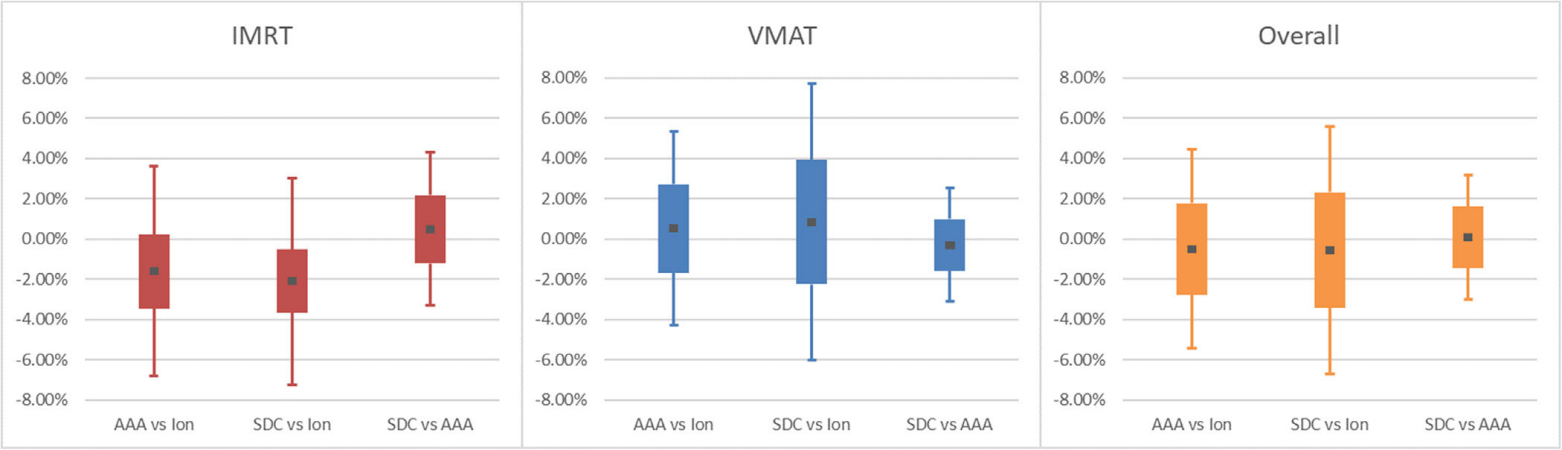
Box and whisker representation of the table above. Average value is denoted by the dark central dot. Solid bar represents standard deviation, and confidence limits (CLs) are represented graphically by the upper and lower whiskers

**FIGURE 3:**
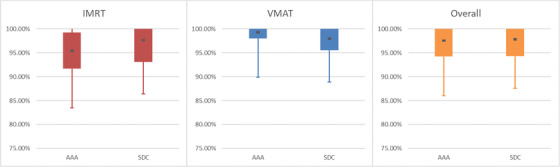
Graphical representation of Table 6. Analytic Anisotropic Algorithm (AAA) results and Sun Nuclear Dose Calculator (SDC) results are placed side by side. Average is the dark central dot. Solid bar represents standard deviation. Confidence limits (CLs) are represented graphically by the whiskers. Note that gamma analysis does not allow for a standard two‐tailed Gaussian distribution as it caps at 100%

**FIGURE 4:**
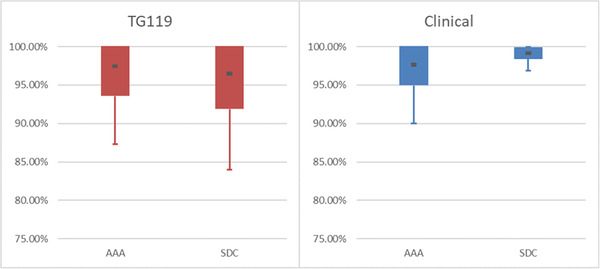
Graph of Table 7 data. Of particular note is how the relative passing rates differ between each plan population. Analytic Anisotropic Algorithm (AAA) matched TG119 delivery better than Sun Nuclear Dose Calculator (SDC), while SDC matched the clinical plan delivery better than AAA. Reference to the overall data can be seen in Figure 3/Table 6

For the 6FFF beam at our pilot site, ion chamber and film measurements were performed following TG‐119 recommendations in a 30 × 30 cm^2^, 20 cm deep solid water phantom. Table 5 and Figure 2 below summarize point measurements taken at isocenter as well as a shifted anterior and/or posterior depending on the positions of relevant OARs for the dataset (e.g., rectum for a prostate‐type plan). Table 6 and Figure 3, along with Table 7 and Figure 4 below summarize the results for the film measurements, categorizing the analysis by delivery type (IMRT vs. Volumetric Modulated Arc Therapy (VMAT)) and plan population (TG‐119 vs. clinical). Gamma criteria used were 3%/3 mm 10% threshold for conventional beams and 3%/1 mm 10% threshold for SRS beams. One of the goals of TG‐119 was to provide baseline CL that other facilities can use to inform their commissioning. It states that “Locally derived CL that substantially exceed these baseline values may indicate the need for improved IMRT Commissioning.”^14^ For ion chamber measurements TG‐119 Tables VII, VIII, IX and X^14^ show CLs ranging from 1.5% to 9.8% with a mean CL of approximately 4.5% (or 0.045) across all institutions surveyed. The 6FFF ion chamber comparisons presented in this work showed CLs of 4.95% for AAA and 6.15% for SDC. This was deemed acceptable due to the robustness of the film analysis. For film measurements, TG119 Table XI^14^ shows CL of 12.4% for 3%/3 mm gamma criteria. It was the goal then to minimally be within these local CL for all machines. Notably, for film this was achieved even for 3%/1 mm criteria in most cases (see 6FFF, Table 6).

**TABLE 5:** 6FFF ion chamber measurement agreement with Analytic Anisotropic Algorithm (AAA) and, Sun Nuclear Dose Calculator (SDC), as well as a point dose comparison between AAA and SDC. Analysis is separated by delivery technique and overall. Confidence limits (CLs) were computed for 95% confidence interval (1.96σ)

	IMRT			VMAT			Overall		
Analysis criteria	AAA vs. Ion	SDC vs. Ion	SDC vs. AAA	AAA vs. Ion	SDC vs. Ion	SDC vs. AAA	AAA vs. Ion	SDC vs. Ion	SDC vs. AAA
%Difference									
Avg	−1.60%	−2.08%	0.50%	0.52%	0.84%	−0.29%	−0.49%	−0.55%	0.08%
Std Dev	1.84%	1.56%	1.69%	2.19%	3.08%	1.28%	2.28%	2.86%	1.52%
CL	5.21%	5.14%	3.82%	4.81%	6.87%	2.80%	4.95%	6.15%	3.07%

Abbreviation: VMAT, volumetric modulated arc therapy.

**TABLE 6:** 6FFF film analysis for the TG119 and clinical plan populations used for commissioning Sun Nuclear Dose Calculator (SDC). Criterion used was 3%/1 mm (srs beam). Analysis was also divided out by static gantry IMRT, VMAT, and overall plan population. Confidence limits (CLs) were computed for 95% confidence interval (1.96σ)

Analysis criteria	IMRT		VMAT		Overall	
	AAA	SDC	AAA	SDC	AAA	SDC
3%/1 mm Gamma						
Avg	95.46%	97.63%	99.30%	98.01%	97.54%	97.83%
Std Dev	3.79%	4.52%	1.26%	2.44%	3.30%	3.52%
CL	11.96%	11.23%	3.17%	6.78%	8.93%	9.07%

Abbreviation: AAA, Analytic Anisotropic Algorithm; VMAT, Volumetric Modulated Arc Therapy.

**TABLE 7:** 6FFF analysis of film passing rates separated by TG119 plans and clinical plans. Criterion used was 3%/1 mm (srs beam)

	TG119		Clinical	
Analysis criteria	AAA	SDC	AAA	SDC
3%/1 mm Gamma				
Avg	97.47%	96.51%	97.62%	99.15%
Std Dev	3.89%	4.61%	2.66%	0.74%
CL	10.16%	12.54%	7.60%	2.29%

Abbreviations: AAA, Analytic Anisotropic Algorithm; CL, confidence limit; SDC, Sun Nuclear Dose Calculator.

Separating analysis via delivery type (IMRT vs. VMAT, Tables 5 and 6, Figures 2 and 3) or plan population (TG119 vs. Clinical, Table 7, Figure 4) may be instructive in helping to set model optimization goals. It can also help with expected agreement for special cases when using delivery techniques that are emphasized more, or not employed as often.

**FIGURE 5:**
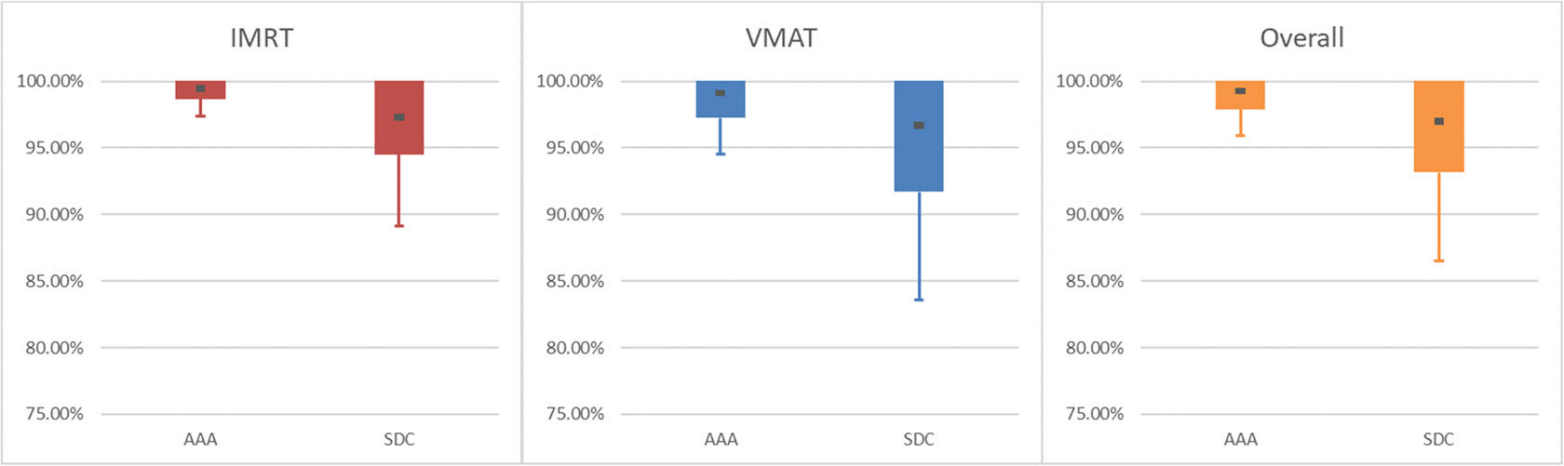
Graph of Table 8. Note that confidence limit (CL) for overall plan population for Sun Nuclear Dose Calculator (SDC) was 10.52% (difference between central dot and bottom whisker). This was deemed unacceptable and further refinements were requested

**FIGURE 6:**
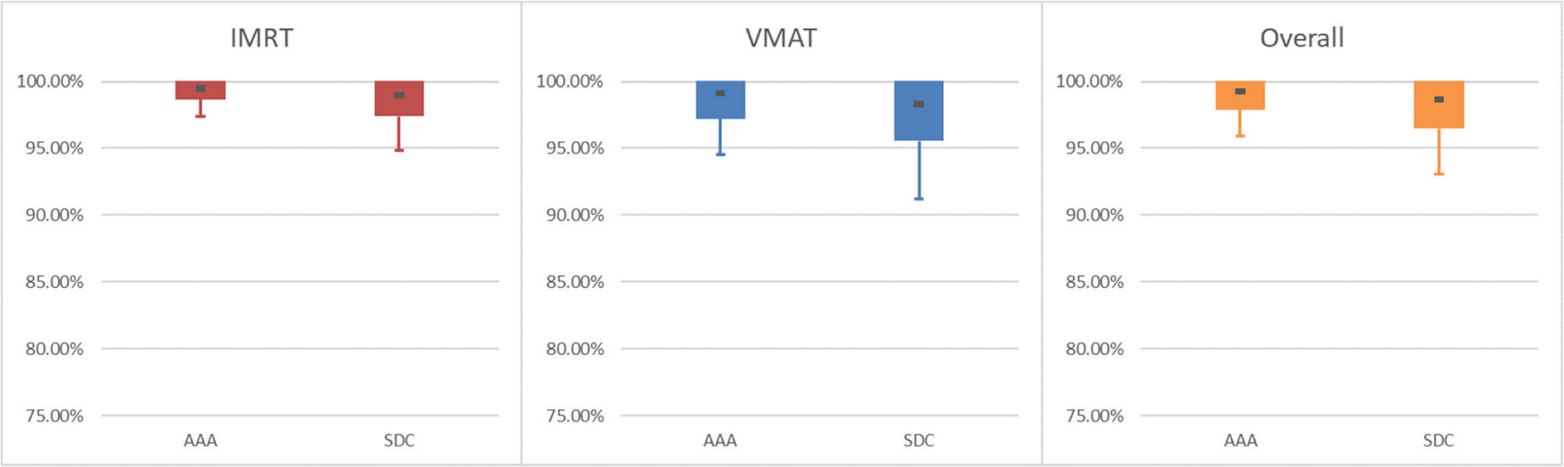
Graph of Table 9. Note that improvements can be seen for both delivery techniques and the confidence limit (CL) for overall plan population for Sun Nuclear Dose Calculator (SDC) improved to 5.58%

For the flattened energies (6x, 10x) similar analysis was performed. In the case of 6x, the initial model was noted to be deficient (Table 8 and Figure 5). A small adjustment to the tongue‐and‐groove parameter and a larger adjustment to the leaf gap parameter were performed by the vendor using guidance from TG‐119 plans (film and chamber measurements) and additional patient datasets. The MLC modeling tweaks for this energy improved film analysis results (shown in Table 9 and Figure 6).

**TABLE 8:** 6x initial model film analysis. Gamma analysis criterion used was 3%/3 mm (conventional beams)

	IMRT		VMAT		Overall	
Analysis criteria	AAA	SDC	AAA	SDC	AAA	SDC
3%/3 mm Gamma						
Avg	99.46%	97.29%	99.11%	96.71%	99.28%	97.00%
Std Dev	0.77%	2.78%	1.88%	5.02%	1.37%	3.84%
CL	2.06%	8.17%	4.57%	13.12%	3.40%	10.52%

Abbreviations: AAA, Analytic Anisotropic Algorithm; CL, confidence limit; SDC, Sun Nuclear Dose Calculator; VMAT, Volumetric Modulated Arc Therapy.

**TABLE 9:** 6x updated model film analysis. Gamma analysis criteria used was 3% 3 mm (conventional beams)

	IMRT		VMAT		Overall	
Analysis criteria	AAA	SDC	AAA	SDC	AAA	SDC
3%/3 mm Gamma						
Avg	99.46%	98.97%	99.11%	98.32%	99.28%	98.65%
Std Dev	0.77%	1.58%	1.88%	2.78%	1.37%	2.16%
CL	2.06%	4.12%	4.57%	7.12%	3.40%	5.58%

Abbreviations: AAA, Analytic Anisotropic Algorithm; CL, confidence limit; SDC, Sun Nuclear Dose Calculator; VMAT, Volumetric Modulated Arc Therapy.

### 3.3 Digital comparison result

**FIGURE 7:**
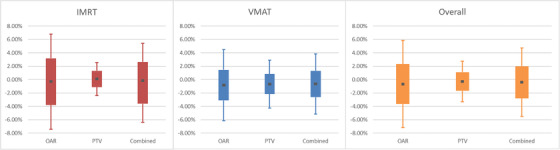
Graph of Table 10. The agreement is notably tighter for target structures than organs at risk

**FIGURE 8:**
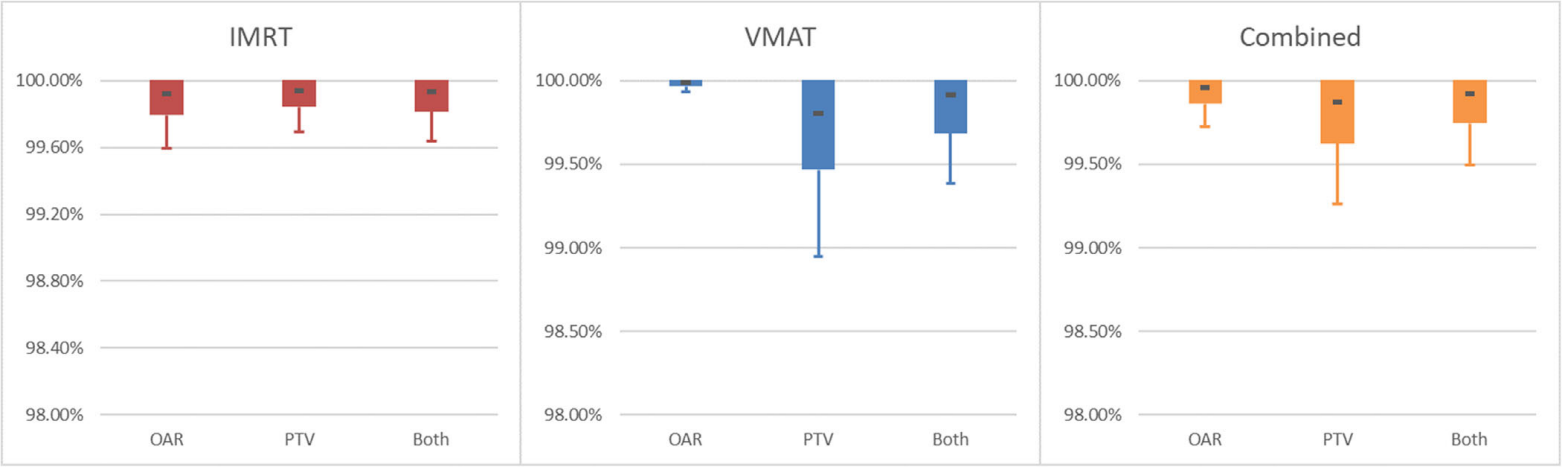
Graph of Table 11. Note the change in the *Y*‐axis scale from the film gamma analysis. The smaller scale was used to help show differences between the modalities and structure types

After establishing model agreement to measurement, the next goal was to establish a baseline of expected agreement between AAA and SDC using the metrics employed by DoseCHECK: clinical DVH metrics (Table 10 and Figure 7) and 3D gamma rates (Table 11 and Figure 8). TG‐119 plans were used to establish agreement in a water phantom environment. The analysis included an examination of OARs, Planning Target Volumes (PTVs) and all structures combined and included IMRT and VMAT delivery. For 3D gamma, global normalization was used for 3%/3 mm (conventional) and 3%/1 mm criteria (srs).

**TABLE 10:** 6FFF AAA vs. SDC DVH point agreement for TG119 plans

	IMRT			VMAT			Overall		
Analysis Criteria	OAR	PTV	Combined	OAR	PTV	Combined	OAR	PTV	Combined
% Difference									
Avg	−0.29%	0.09%	−0.13%	−0.84%	−0.67%	−0.66%	−0.67%	−0.29%	−0.40%
Std Dev	3.48%	1.21%	2.76%	2.28%	1.49%	1.96%	2.99%	1.39%	2.40%
CL	7.10%	2.46%	5.55%	5.31%	3.58%	4.50%	6.52%	3.02%	5.10%

Abbreviations: AAA, Analytic Anisotropic Algorithm; CL, confidence limit; DVH, dose–volume histogram; OAR, Organ at Risk; PTV, Planning Target Volume; SDC, Sun Nuclear Dose Calculator; VMAT, Volumetric Modulated Arc Therapy.

**TABLE 11:** 6FFF AAA vs. SDC 3D Gamma agreement for TG119 plans. Gamma criteria 3%/1 mm (srs beams)

	IMRT			VMAT			Combined		
Analysis criteria	OAR	PTV	Both	OAR	PTV	Both	OAR	PTV	Both
3%/1 mm Gamma									
Avg	99.93%	99.94%	99.93%	99.99%	99.80%	99.91%	99.96%	99.87%	99.92%
Std Dev	0.13%	0.10%	0.12%	0.03%	0.33%	0.23%	0.10%	0.25%	0.18%
CL	0.33%	0.25%	0.29%	0.06%	0.85%	0.53%	0.23%	0.61%	0.42%

Abbreviations: AAA, Analytic Anisotropic Algorithm; CL, confidence limit; OAR, Organ at Risk; PTV, Planning Target Volume; SDC, Sun Nuclear Dose Calculator; VMAT, Volumetric Modulated Arc Therapy.

For desired digital agreement between SDC and AAA, there are various machine setups presented in the Appendix (Tables A1, A2, B1, B2, B3): all machines/energies achieved a combined DVH point agreement CL of 7.47% or below, while the highest combined CLs for 3D gamma criteria of 3%/3 mm (conventional beams), 3%/1 mm (stereotactic beams) was 0.36% and 0.30%, respectively. These reported CLs should only be taken for what was achievable with our commissioning and used as a reference for comparison for your institutions own independent verification. The machine setups presented in the Appendix are energies of 6x, 6FFF, 6SRS, 10x, and 10FFF on Linear Accelerator (CLINAC) and TrueBeam platforms; aggregate data of Millenium MLC and HD MLC machines are provided.

## 4 DISCUSSION

Two main questions come to mind when employing a novel and advanced second check system with a wide range of 3D analysis tools. One is, “How does one achieve a good model?” and the other being “What action limits do I set for the new 3D metrics this system employs?”. To the former, there is a wide array of modeling parameters that can be adjusted to help the model match the desired physical measurements (sweeping gap and TG‐119 ion chamber measurement, TG‐119 film measurements) and digital comparisons (TG‐53, TG‐119 plan comparisons) that have been presented in this document. It should be noted that the purpose of the 2D film analysis was to establish confidence in each AAA and SDC model and assert their comparability under controlled measurable conditions. It is known that 2D gamma pass rates are not directly reflective of 3D gamma pass rates,^17^, ^18^, ^19^ but knowing that the models are comparable in 1D and 2D measurement conditions allows for confidence in studying their agreement in a 3D calculation environment. To the latter question above, once the two models have been validated to the satisfaction of the user, the user should undergo a digital comparison study that focuses on the metrics that the system employs. In this body of work, such metrics include structure‐specific 3D gamma comparisons with TG‐119 plans and patient plans on a phantom and DVH metric comparisons for TG‐119 plans. This study will give average, standard deviation, and CL data that can be used to set action limits for your second check results. As an example, based on digital comparison studies you might expect 95% of your plans to match the PTV D95 within 3%, so it might be logical to set a tolerance of 3% for PTV D95 for your plans. The institution setting the tolerance may elect to set tighter or looser tolerances based on the desired sensitivity or specificity but the digital study will help inform that decision.

This document has provided average, standard deviation, and CL data for a variety of machines as a guide to the experience within the Henry Ford Health System. These data should not be used to set tolerances/action limits at other institutions but can be used as a guide for what is achievable at the time of commissioning.

### 4.1 Experiences regarding modeling with SDC

One of the difficulties of presenting on the experiences of commissioning this system is the difficulty of presenting data on the numerous intermediary modeling iterations that can show how various problems and shortcomings may have been overcome. Here, we will discuss our experience with different aspects of model tuning, to demonstrate a couple of direct scenarios where models were determined to be deficient. Insight into the direction that modeling efforts should take, and the potential trade‐offs at various steps, will help streamline the modeling and model validation process. The final iterations of the models for various machines are presented in the body and Appendix of this text.

**FIGURE 9:**
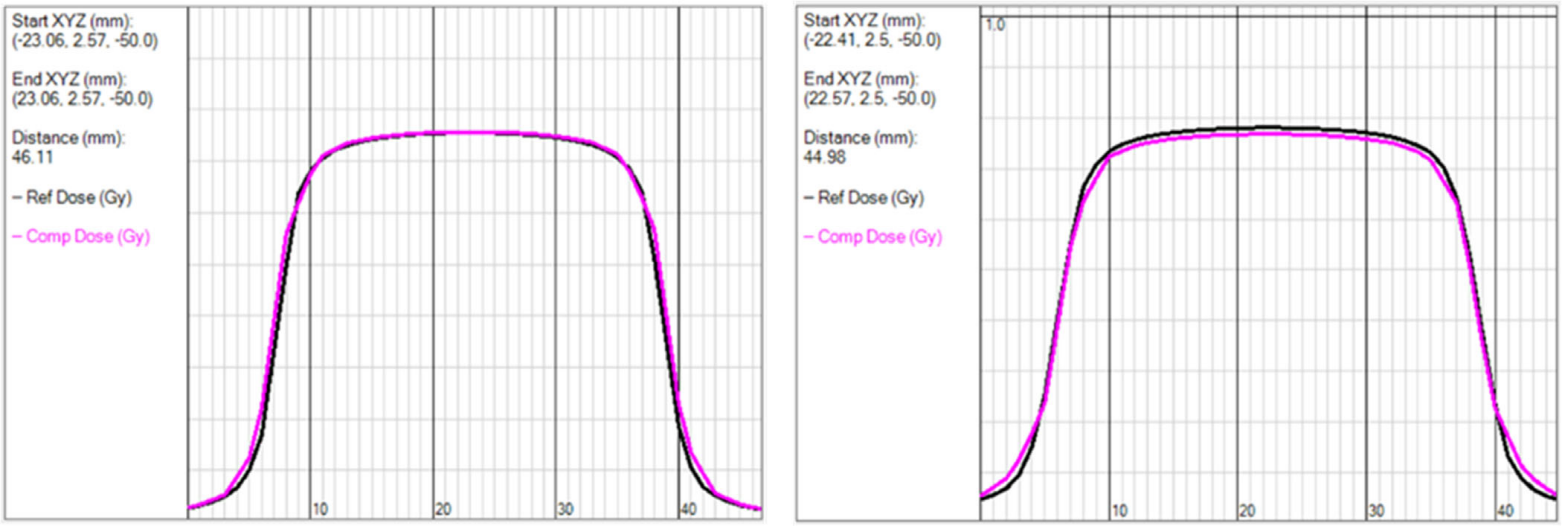
Overlay of calculated doses for 3 × 3 Jaw (left) and 3 × 3 MLC (right) fields for Analytic Anisotropic Algorithm (AAA) (dark blue) and Sun Nuclear Dose Calculator (SDC) (pink). Note slight changes in agreement near the shoulders and in the penumbral tails between the systems in the MLC bound situation

One of the first evaluation points was TG‐53 square fields shaped by both the Jaws and MLC. IAEA report TRS 483 has noted that small fields can be more affected by MLC uncertainties (such as MLC calibration issues) than larger fields. This puts further emphasis on the accuracy of MLC modeling.^20^ Understanding how the MLC modeling in SDC and AAA compares to each other is critical as they model the MLC in different ways which can impact small field scenarios (Figure 9). This can be interesting as small fields (below 5 × 5 cm^2^) are known to be difficult to characterize^21^ due to focal source occlusion and overlapping penumbra.^20^ The extra‐focal source (collimator scatter source) is also significantly occluded and factors into the characteristics of these small field scenarios. In the case of MLC fields, adding in jaw settings that differ greatly (20 × 20 cm^2^ jaw setting for 5 × 5 cm^2^ MLC field sizes and below) from the MLC shape size adds further complexity the models must account for. In some modeling systems (SDC and Pinnacle), the modeling of the extra focal source determines the model predicted output factors which are determined by the jaw settings.^10^ If these output factors are different from the measured output factors, then a correction is applied to help correct the model to the measured values. The jaw setting is used by the model to help determine both the predicted output factor and the output factor correction. Creating small field sizes with MLCs with a large jaw setting at 20 × 20 cm^2^ means that most of the source occlusion, output changes (extra focal source occlusion), or other small field effects are now due to the MLC (the tertiary collimator) instead of the jaw. There is potential then for the output correction to be inaccurate or misapplied. This scenario essentially tests how well the extra focal source is being modeled. This can be clinically relevant since VMAT plans with large targets will often have larger jaw settings (10 × 10 cm^2^ or greater) but small MLC segments modulating the beam.^22^ The points where the digital agreement between SDC and AAA exceeded TG‐53 recommendations were in instances where the most complex of modeling situations were seen (small MLC bound square fields) and the models showed consistency with each other down to about the 2 × 2 cm^2^ MLC field size. The two lowest of MLC field sizes (1 × 1 and 1.5 × 1.5 cm^2^) were where consistent disagreement was observed and similar disagreement has been noted between AAA and Acuros XB in the literature (Kron et al., Table II).^22^, ^23^ It was determined that the similarities seen between AAA and SDC in the majority of the stressful modeling situations was acceptable.

When it came to more complex plan delivery we started with the TG‐119 recommendation to ensure our measured film analysis CL to be at least within the local CL reported in TG‐119. There were notable instances where this was not achieved on the first model iteration, the 6x beam on the pilot machine is one such instance. Table 8 (film analysis for the 6x beam) notes that the VMAT delivery exceeds the average local CL reported by TG‐119 of 12.4%. Since VMAT is our main mode of delivery it was determined that this model required further revision. Additional plans were provided to the vendor who fine‐tuned the MLC model based on these additional plans to achieve a better match. Based on this instance, we think it is good to have part of the analysis based on delivery type to ensure no outstanding issues are occurring for a particular delivery type. Ideally both delivery types will be found to be acceptable, but knowing where tradeoffs can occur will help in tailoring the model to your institutional needs.

It has been noted in the literature that some models may be tuned for small or large field delivery based on their main modality (e.g., SRS vs. conventional) for delivery.^24^ This focused tuning of the model can improve agreement with delivered measurements for some modeling systems, especially those employing a DLG value for modeling purposes.^24^, ^25^ During the optimization of the models in SDC, it was noted that easy and hard C shapes are the most problematic plans to deliver accurately. These TG‐119 plans closely resemble stereotactic spine plans that often have small fields and high modulation. Larger TG‐119 plans such as the head and neck, prostate, or multi‐target had lower incidence of disagreement with measurement. So, when we were selecting plans to help guide model tuning, we preferentially used the C‐shaped plans as reference. A good example of this in action is experience with our Varian Edge model which is elaborated below. Models that were able to adequately deliver easy and hard C‐shaped plans were typically able to deliver the other TG‐119 plan types. Whereas some models that were able to deliver the other plan types exhibited lower passing rates for the C‐shaped plans. In general, it seemed that tuning the SDC models for the smaller field higher modulation plans would not significantly negatively impact larger field plans, whereas the reverse scenario was not as certain to yield good passing results for all plan types.

Analysis of the data can be organized in multiple ways to offer potential insights for clinical use. In this case, we separated out different delivery modes (IMRT vs. VMAT) and different planning populations (clinical vs. TG119). For the pilot site 6FFF beam, we notice variation in CL based on delivery technique (Table 6). IMRT 3%/1 mm CL was about 11% for both AAA and SDC, while for VMAT CL fell to about 3% for AAA and 7% for SDC. Our system overwhelmingly employs VMAT techniques over static gantry IMRT so attempting to improve the result of IMRT to the detriment of VMAT deliveries would not be desirable (though results for both techniques are within acceptable ranges).

Differences in CL were also noticed when separating analysis via plan population (Table 7). For the same beam as above, SDC had a lower CL than AAA for clinical plans (3%/1 mm CL of 7.60% AAA vs. 2.29% SDC), while for the TG119 population AAA achieved a lower CL limit than SDC (3%/1 mm CL of 10.16% AAA vs. 12.54% SDC). This is interesting to note as the plan types involved in the TG119 test suite may not be as applicable to the patient population that you intend on treating. It seems that plan populations might stress the models in different ways. TG‐119 might have a wider variety of plan types, while the patient population you may be primarily treating may be more of a smaller field type. Thus, there is good reason to include a subset of clinically delivered plans to help accurately evaluate beams that are used for high‐risk specialty treatments like SRS/SBRT. These results for the 6FFF beam might not be that surprising upon reflection as the C‐shaped plans were very instructive in fine tuning our models and these C shapes are also more reflective of the SRS/SBRT plan types that are typically used on the unflattened beams.

There were also some instances where even though the baseline sought after agreement with TG‐119 plans was achieved we desired more stringent agreement. The most obvious case of this was with our Varian Edge machine. In this case, sweeping gap calculations and sweeping gap measurements were provided to Sun Nuclear from the start as our edge model was one of their first HDMLC models. The sweeping gap data helped with understanding the leaf trajectories for the HDMLC machines. During the evaluation process, it was noticed that the SDC model was predicting the dose to be colder on a TG‐119 C‐shaped plan than both AAA and the film measurement. After evaluation of other TG‐119 and patient plans, it was determined to increase the leaf transmission and leaf‐offset parameter and decrease the leaf‐gap parameter using the C‐shaped plan as a guide. This meant that the MLC model parameters would be moved off the values established by the sweeping gap calculations and measurements. The modelers were instructed to find a “midpoint” between MLC parameters that best fit the sweeping gaps and those that best fit the C‐shaped plan. Additionally, the tongue and groove thickness parameter was tweaked to aid in matching verified VMAT dose distributions. As can be seen in the results for the Edge machine in the Appendix (Table A1), these compromises and tweaks during the modeling process resulted in SDC providing better film agreement than our primary clinical AAA model for a variety of film comparisons that included both clinical patient plans and TG‐119 plans.

### 4.2 Setting agreement criteria and action limits

Both SDC and AAA have achieved TG‐119 and TG‐53 standard of agreement with measurement and now we can assess their probability of agreement with each other in a clinically useful manner. For instance, we can take a clinically relevant metric (e.g., D95) and be able to ascertain a probability that any given SDC calculation will agree with the primary TPS calculation and set action limits accordingly.

From the TG‐119 plans, DVH point (Table 10) and 3D gamma agreement (Table 11) were analyzed to establish a baseline of agreement. For the 6FFF beam, the DVH point agreement was –0.40% ± 2.4% (CL: 5.1%) and the 3D Gamma (3%/1 mm) 99.92% avg. 0.18% SD (CL: 0.42%). These local CL can be useful when setting clinical action limits for the various programs in a center. In the case of the Stereotactic beams, it was determined that target D95 was a useful metric to examine. The DVH point CLs for the various stereotactic beams in the system indicated that 95% of DVH points should be within 5% agreement. Accordingly, we set action criteria of D95 agreement of ± 5% for further investigation or review (each case will have accompanying PSQA measurements performed in addition to a secondary dose calculation).

SNC also has the option to use local normalization for the 3D Gamma analysis. Though these numbers are not included in this paper our internal assessment was that the increased sensitivity may be of use for structure or volumes that contain high‐dose gradients. However, local normalization will also trigger points of failure in low‐dose regions that lead to no clinical consequence. This will often give the body contour an artificially low pass rate. Thus, it is not currently being employed.

It should be noted that we are not indicating that any SDC model will give a D95 within 5%/95% of the time. Rather, each institution, after accepting and validating an SDC model, can use this method to help inform them about what criteria they should use and what actionable situations will be. The analysis and data here can serve as a guide for what is potentially achievable but should not be used directly. Each institution should undergo their own study to acquire relevant local data to inform their clinical actionable criteria.

Lastly, evaluation of dose accuracy in the case of tissue heterogeneity (e.g., small lung tumors) is an important component of overall commissioning of the secondary check system. The algorithm employed by the TPS calculations is based on the AAA algorithm. We extensively studied the differences between the AAA and superposition convolution (S/C) and other algorithms (including pencil beam, Acuros, Monte Carlo) for a large cohort of early‐stage lung cancer patients^26^ and showed that differences between S/C and AAA algorithms for small lung tumors were within 2%–3% on average. However, the implementation of an S/C algorithm can vary among different systems, so it is important that the accuracy be tested for the specific secondary check system. We intend to evaluate the heterogeneity differences for this secondary check system incorporating confidence (CI) limits as well, as part of future comprehensive investigation.

### 4.3 Recommended process for model validation

This is a short list for the general steps for creating a good model and establishing baseline agreement between the TPS and dose check. This process assumes an already well‐commissioned primary TPS, which allows some digital comparisons in a homogeneous water‐like environment with the TPS to quickly eliminate gross errors in the SDC model at some stages.

1. Spot check various regions of open jaw and MLC fields as recommended by TG‐53.
2. Develop IMRT and VMAT plans in a solid water phantom via recommendations from TG‐119.
3. Perform a preliminary digital comparison between DoseCHECK (SDC) and your primary TPS focusing on target agreement. This will help highlight gross modeling errors in clinical situations.
4. Perform ion chamber measurements (and film if possible) to verify the SDC model against measurement. Compare to primary TPS agreement with measurement for the same plans. This will serve as a guide to either confirm a problematic trend in the SDC model that should be corrected or to establish confidence in the SDC calculation.
5. If disagreement is noticed, you may contact Sun Nuclear about further optimizing the SDC model. Further data for the fine tuning may be provided. The necessary data will depend on the area of emphasis for this particular model.
6. After updated SDC models have achieved sufficient agreement with measurements, perform an evaluation of target and OAR agreement with the primary TPS using the TG‐119 plans to establish a baseline of agreement in solid water. This is to help inform clinical action limits.

One may use the data in this document as a guide to compare their results to the models for their machines. Provided in the Appendix are the results from various machines/energies commissioned at the Henry Ford Health System (Tables A1, A2, B1, B2, B3). Machine platforms include CLINAC and TrueBeam/Edge each with an example of both a Millenium MLC and an HD MLC.

## 5 CONCLUSIONS

The Sun Nuclear Dose Check second check package provides 3D comparison tools, such as 3D gamma and DVH point comparisons, allowing for an in‐depth means of plan evaluation that was not previously readily available in standard clinics today. The rigor of these comparison tools is bolstered by the sophistication of the CCC algorithm that it employs. This system is a large leap forward over the previous standard of point dose comparisons using effective path length algorithms. However, with this increased complexity also comes an additional burden of rigor of model commissioning and evaluation of clinically significant action levels for the new evaluation tools employed. It has been established in this document that the modeling done by these new 3D second check systems can achieve agreement with measurements within the TG‐119 CL. SDC was able to match the primary TPS in digital agreement within parameters outlined by TG‐53. In a side‐by‐side comparison using TG‐119 for guidance, SDC showed the capability of matching AAA in agreement with measured film and ion chamber results. Having established that measurements were within TG‐119 recommendations, baselines for 3D gamma pass rates (target and OAR) and DVH statistics were gathered from the solid water plans employed by TG‐119 and were used to inform clinically relevant action levels.

## CONFLICT OF INTEREST

The authors declare that there is no conflict of interest that could be perceived as prejudicing the impartiality of the research reported.

## AUTHOR CONTRIBUTIONS

Brian Bismack created the models, test plans, and lead the data analysis. Jennifer Dolan, Eric Laugeman, and Anant Gopal helped acquire measurements, analyze film measurements, and data analysis. Ning Wen and Indrin Chetty contributed to research design and data analysis.

## A Cumulative data for HD MLC machines

**TABLE A1:** High‐dose rate beam (6FFF/6srs) HD MLC machine results, film results include clinical plan and TG119 plan results, note 3%/1 mm gamma criteria for SRS beams

Film						
	IMRT		VMAT		Combined	
Analysis criteria	AAA	SDC	AAA	SDC	AAA	SDC
3%/1 mm Gamma						
Avg	97.28%	97.90%	98.58%	99.19%	98.14%	98.76%
Std Dev	3.74%	3.71%	2.64%	0.97%	3.05%	2.30%
CL	10.05%	9.38%	6.60%	2.70%	7.84%	5.74%

DVH									
	IMRT			VMAT			Combined		
Analysis Criteria	OAR	PTV	Both	OAR	PTV	Both	OAR	PTV	Both
% Difference									
Avg	1.43%	0.83%	1.18%	0.98%	0.44%	0.76%	1.22%	0.64%	0.97%
Std Dev	1.96%	1.11%	1.69%	1.97%	0.77%	1.56%	1.97%	0.97%	1.64%
CL	5.28%	3.01%	4.49%	4.85%	1.94%	3.82%	5.09%	2.53%	4.18%

3D Gamma									
	IMRT			VMAT			Combined		
Analysis criteria	OAR	PTV	Both	OAR	PTV	Both	OAR	PTV	Both
3%/1 mm Gamma									
Avg	99.97%	99.92%	99.95%	99.99%	100.00%	99.99%	99.98%	99.96%	99.97%
Std Dev	0.12%	0.26%	0.19%	0.05%	0.01%	0.04%	0.09%	0.18%	0.14%
CL	0.27%	0.59%	0.42%	0.10%	0.02%	0.08%	0.20%	0.40%	0.30%

Abbreviations: AAA, Analytic Anisotropic Algorithm; CL, confidence limit; DVH, dose–volume histogram; MLC, Multileaf Collimator; OAR, Organ at Risk; PTV, Planning Target Volume; SDC, Sun Nuclear Dose Calculator; SRS, Stereotactic Radiosurgery; VMAT, Volumetric Modulated Arc Therapy.

**TABLE A2:** Conventional 6x beam HD MLC results, note 3%/3 mm gamma criteria for conventional beams

Film						
	IMRT		VMAT		Combined	
Analysis Criteria	AAA	SDC	AAA	SDC	AAA	SDC
3%/3 mm Gamma						
Avg	98.64%	99.76%	99.82%	99.85%	99.20%	99.80%
Std Dev	2.19%	0.40%	0.43%	0.23%	1.68%	0.32%
CL	5.64%	1.02%	1.02%	0.59%	4.10%	0.83%

DVH									
	IMRT			VMAT			Combined		
Analysis Criteria	OAR	PTV	Both	OAR	PTV	Both	OAR	PTV	Both
% Difference									
Avg	1.93%	0.61%	1.39%	1.22%	−0.01%	0.71%	1.57%	0.30%	1.05%
Std Dev	2.76%	1.14%	2.32%	2.09%	1.09%	1.84%	2.46%	1.15%	2.12%
CL	7.33%	2.85%	5.94%	5.31%	2.15%	4.33%	6.40%	2.56%	5.20%

3D Gamma									
	IMRT			VMAT			Combined		
Analysis criteria	OAR	PTV	Both	OAR	PTV	Both	OAR	PTV	Both
3%/3 mm Gamma									
Avg	100.00%	100.00%	100.00%	100.00%	99.99%	99.99%	100.00%	99.99%	100.00%
Std Dev	0.00%	0.00%	0.00%	0.00%	0.05%	0.03%	0.00%	0.04%	0.02%
CL	0.00%	0.00%	0.00%	0.00%	0.12%	0.07%	0.00%	0.08%	0.05%

Abbreviations: AAA, Analytic Anisotropic Algorithm; CL, confidence limit; DVH, dose–volume histogram; MLC, Multileaf Collimator; OAR, Organ at Risk; PTV, Planning Target Volume; SDC, Sun Nuclear Dose Calculator; VMAT, Volumetric Modulated Arc Therapy.

## B Cumulative data for Millennium MLC machines

**TABLE B1:** 6FFF Millennium MLC Machine results, film results include Clinical Plan and TG119 plan results

Film						
	IMRT		VMAT		Combined	
Analysis criteria	AAA	SDC	AAA	SDC	AAA	SDC
3%/1 mm Gamma						
Avg	96.86%	98.34%	99.24%	98.10%	98.26%	98.20%
Std Dev	3.72%	3.46%	1.43%	2.94%	3.33%	3.14%
CL	10.42%	8.44%	3.57%	7.67%	8.26%	7.96%

DVH									
	IMRT			VMAT			Combined		
Analysis criteria	OAR	PTV	Both	OAR	PTV	Both	OAR	PTV	Both
% Difference									
Avg	0.94%	0.80%	0.88%	0.94%	0.32%	0.65%	0.94%	0.56%	0.77%
Std Dev	3.09%	1.22%	2.49%	2.83%	1.43%	2.30%	2.96%	1.34%	2.40%
CL	6.99%	3.19%	5.76%	6.49%	3.11%	5.17%	6.74%	3.19%	5.47%

3D Gamma									
	IMRT			VMAT			Combined		
Analysis criteria	OAR	PTV	Both	OAR	PTV	Both	OAR	PTV	Both
3%/1 mm Gamma									
Avg	99.98%	99.91%	99.96%	99.98%	99.94%	99.97%	99.98%	99.94%	99.97%
Std Dev	0.06%	0.24%	0.14%	0.05%	0.18%	0.12%	0.06%	0.19%	0.13%
CL	0.14%	0.55%	0.31%	0.12%	0.41%	0.27%	0.14%	0.43%	0.29%

Abbreviations: AAA, Analytic Anisotropic Algorithm; CL, confidence limit; DVH, dose–volume histogram; MLC, Multileaf Collimator; OAR, Organ at Risk; PTV, Planning Target Volume; SDC, Sun Nuclear Dose Calculator; VMAT, Volumetric Modulated Arc Therapy.

**TABLE B2:** 6x millennium MLC machine results

Film						
	IMRT		VMAT		Combined	
Analysis criteria	AAA	SDC	AAA	SDC	AAA	SDC
3%/3 mm Gamma						
Avg	99.66%	99.57%	99.57%	99.49%	99.62%	99.53%
Std Dev	0.67%	0.90%	1.10%	1.36%	0.90%	1.14%
CL	1.64%	2.20%	2.58%	3.17%	2.15%	2.71%

DVH									
	IMRT			VMAT			Combined		
Analysis criteria	OAR	PTV	Both	OAR	PTV	Both	OAR	PTV	Both
% Difference									
Avg	2.33%	0.09%	1.41%	0.28%	0.09%	0.20%	1.34%	0.09%	0.80%
Std Dev	4.87%	1.18%	3.96%	3.24%	1.55%	2.60%	4.28%	1.37%	3.40%
CL	11.87%	2.39%	9.17%	6.64%	3.12%	5.29%	9.73%	2.77%	7.47%

3D Gamma									
	IMRT			VMAT			Combined		
Analysis criteria	OAR	PTV	Both	OAR	PTV	Both	OAR	PTV	Both
3%/3 mm Gamma									
Avg	100.00%	99.92%	99.97%	100.00%	99.95%	99.98%	100.00%	99.93%	99.97%
Std Dev	0.00%	0.34%	0.22%	0.00%	0.15%	0.10%	0.00%	0.26%	0.17%
CL	0.00%	0.75%	0.47%	0.00%	0.35%	0.22%	0.00%	0.58%	0.36%

Abbreviations: AAA, Analytic Anisotropic Algorithm; CL, confidence limit; DVH, dose–volume histogram; MLC, Multileaf Collimator; OAR, Organ at Risk; PTV, Planning Target Volume; SDC, Sun Nuclear Dose Calculator; VMAT, Volumetric Modulated Arc Therapy.

**TABLE B3:** 10x millennium MLC machine results

Film						
	IMRT		VMAT		Combined	
Analysis criteria	AAA	SDC	AAA	SDC	AAA	SDC
3%/3 mm Gamma						
Avg	99.90%	99.75%	99.76%	99.62%	99.83%	99.69%
Std Dev	0.22%	0.62%	0.59%	1.00%	0.44%	0.82%
CL	0.54%	1.45%	1.40%	2.33%	1.04%	1.91%

DVH									
	IMRT			VMAT			Combined		
Analysis criteria	OAR	PTV	Both	OAR	PTV	Both	OAR	PTV	Both
% Difference									
Avg	0.85%	−0.54%	0.28%	0.11%	−0.46%	−0.13%	0.50%	−0.50%	0.07%
Std Dev	2.66%	0.93%	2.23%	2.37%	1.71%	2.06%	2.55%	1.37%	2.15%
CL	6.07%	2.37%	4.65%	4.75%	3.81%	4.17%	5.49%	3.19%	4.30%

3D Gamma									
	IMRT			VMAT			Combined		
Analysis criteria	OAR	PTV	Both	OAR	PTV	Both	OAR	PTV	Both
3%/3 mm Gamma									
Avg	100.00%	99.99%	99.99%	100.00%	99.95%	99.98%	100.00%	99.97%	99.99%
Std Dev	0.00%	0.05%	0.03%	0.00%	0.11%	0.07%	0.00%	0.08%	0.06%
CL	0.00%	0.11%	0.07%	0.00%	0.26%	0.16%	0.00%	0.19%	0.12%

Abbreviations: AAA, Analytic Anisotropic Algorithm; CL, confidence limit; DVH, dose–volume histogram; MLC, Multileaf Collimator; OAR, Organ at Risk; PTV, Planning Target Volume; SDC, Sun Nuclear Dose Calculator; VMAT, Volumetric Modulated Arc Therapy.

## References

[1] Nelms B . Methods, software and datasets to verify DVH calculations against analytical values: Twenty years late(r). Med Phys. 2015;42:4435. 10.1118/1.4923175 26233174

[2] Zhen H . Moving from gamma passing rates to patient DVH‐based QA metric in pretreatment dose QA. Med Phys. 2011;38:5477‐5489. 21992366 10.1118/1.3633904

[3] Dempsey JF . A Fourier analysis of the dose grid resolution required for accurate IMRT fluence map optimization. Med Phys. 2005;32:380‐388. 15789583 10.1118/1.1843354

[4] Ebert MA . Comparison of DVH data from multiple radiotherapy treatment planning systems. Phys Med Biol. 2010;55:N337‐N346. 20463378 10.1088/0031-9155/55/11/N04

[5] Nelms B . Evaluating IMRT and VMAT dose accuracy: practical examples of failure to detect systematic errors when applying a commonly used metric and action levels. Med Phys. 2013;40:111722. 24320430 10.1118/1.4826166 PMC8353583

[6] Corporation SN . White Paper: On the Accuracy of the SNC Dose Calculator Algorithm.

[7] Hernandez V . Commissioning of the tongue‐and‐groove modelling in treatment planning systems: From static fields to VMAT treatments. Phys Med Biol. 2017;62:6688‐6707. 28639942 10.1088/1361-6560/aa7b1a

[8] Bailey DW . EPID dosimetry for pretreatment quality assurance with two commercial systems. J Appl Clin Med Phys. 2012;13(4):82‐99. 10.1120/jacmp.v13i4.3736 PMC5716510 22766944

[9] Wen W . Characteristics of a novel treatment system for linear accelerator‐based stereotactic radiosurgery. J Appl Clin Med Phys. 2015;16(4):125‐148. 26218998 10.1120/jacmp.v16i4.5313 PMC5690003

[10] Jacques R . Real‐time dose computation: gPU‐accelerated source modeling and superposition/convolution. Med Phys. 2011;38(1):294‐305. 21361198 10.1118/1.3483785

[11] Hillman Y, Kim J, Chetty I, Wen N . Refinement of MLC modeling improves commercial QA dosimetry system for SRS and SBRT patient‐specific QA. Med Phys. 2018;45:1351‐1359. 29431865 10.1002/mp.12808

[12] Szpala S . On using the dosimetric leaf gap to model the rounded leaf ends in VMAT/RapidArc plans. J Appl Clin Med Phys. 2013;15(2):67‐84. 10.1120/jacmp.v15i2.4484 PMC5875471 24710433

[13] Fraass B . American Association of Physicists in Medicine Radiation Therapy Committee Task Group 53: Quality assurance for clinical radiotherapy treatment planning. Med Phys. 1998;25:1773‐1829. 9800687 10.1118/1.598373

[14] Ezzell GA . IMRT commissioning: Multiple institution planning and dosimetry comparisons, a report from AAPM Task Group 119. Med Phys. 2009;36(11):5359‐5373. 19994544 10.1118/1.3238104

[15] Wen W . Precise film dosimetry for stereotactic radiosurgery and stereotactic body radiotherapy quality assurance using Gafchromic™ EBT3 films. Radiat Oncol. 2016;11. 10.1186/s13014-016-0709-4 PMC5050597 27716323

[16] Kim J‐I . The sensitivity of gamma‐index method to the positioning errors of high‐definition MLC in patient‐specific VMAT QA for SBRT. Radiat Oncol. 2014;9:167. 25070065 10.1186/1748-717X-9-167 PMC4118611

[17] Pulliam KB . Comparison of 2D and 3D gamma analyses. Med Phys. 2016;41:021710‐1‐021710‐6. 10.1118/1.4860195 PMC3977814 24506601

[18] Dhanabalan R, Jeevanandam P, Sukumar P, Ranganathan A, Johnjothi S, Nagarajan V . A study on correlation between 2D and 3D gamma evaluation metrics in patient‐specific quality assurance for VMAT. Med Dosim. 2014;39:300‐308. 24910246 10.1016/j.meddos.2014.05.002

[19] Oldham M , Thomas A , O'Daniel J , et al. A quality assurance method that utilizes 3D dosimetry and facilitates clinical interpretation. Radiat Oncol. 2012;84:540‐546. 10.1016/j.ijrobp.2011.12.015 PMC3832997 22361085

[20] Dosimetry of small static fields used in external beam radiotherapy. International Atomic Energy Agency. 2017; TRS no. 483.

[21] Das IJ . Small fields: Nonequilibrium radiation dosimetry. Med Phys. 2008;38:206‐215. 10.1118/1.2815356 18293576

[22] Kron T . Small field segments surrounded by large areas only shielded by a multileaf collomator: Comparison of experiments and dose calculation. Med Phys. 2012;39(12):7480‐7488. 23231297 10.1118/1.4762564

[23] Fogliata A . Evaluation of the dose calculation accuracy for small fields defined by jaw or MLC for AAA and Acuros XB algorithms. Med Phys. 2016;43(10):5685‐5694. 27782735 10.1118/1.4963219

[24] Yao W . Determining the optimal dosimetric leaf gap setting for rounded leaf‐end multiliaf collimator systems by simple test fields. J Appl Clin Med Phys. 2015;16:65‐77. 10.1120/jacmp.v16i4.5321 PMC5690020 26218999

[25] Kim K . Realtionship between dosimetric leaf gap and dose calculation errors for high definition multi‐leaf collimators in readiotherapy. Phys Imaging Radiat Oncol. 2018;5:31‐36. 33458366 10.1016/j.phro.2018.01.003 PMC7807868

[26] Chetty IJ . Correlation of dose computed using different algorithms with local control following stereotactic ablative radiotherapy (SABR)‐based treatment of non‐small‐cell lung cancer. Radiother Oncol. 2013;109(3):498‐504. 24231237 10.1016/j.radonc.2013.10.012

